# Cavitation erosion by shockwave self-focusing of a single bubble

**DOI:** 10.1016/j.ultsonch.2022.106131

**Published:** 2022-08-22

**Authors:** Fabian Reuter, Carsten Deiter, Claus-Dieter Ohl

**Affiliations:** a Otto-von-Guericke University Magdeburg, Faculty of Natural Sciences, Institute for Physics, Department Soft Matter, Universitaetsplatz 2, Magdeburg 39106, Germany; b European XFEL GmbH, Holzkoppel 4, Schenefeld 22869, Germany

**Keywords:** Cavitation, Cavitation erosion, Shock waves

## Abstract

The ability of cavitation bubbles to effectively focus energy is made responsible for cavitation erosion, traumatic brain injury, and even for catalyse chemical reactions. Yet, the mechanism through which material is eroded remains vague, and the extremely fast and localized dynamics that lead to material damage has not been resolved. Here, we reveal the decisive mechanism that leads to energy focusing during the non-spherical collapse of cavitation bubbles and eventually results to the erosion of hardened metals. We show that a single cavitation bubble at ambient pressure close to a metal surface causes erosion only if a *non-axisymmetric* energy self-focusing is at play. The bubble during its collapse emits shockwaves that under certain conditions converge to a single point where the remaining gas phase is driven to a shockwave-intensified collapse. We resolve the conditions under which this self-focusing enhances the collapse and damages the solid. High-speed imaging of bubble and shock wave dynamics at sub-picosecond exposure times is correlated to the shockwaves recorded with large bandwidth hydrophones. The material damage from several metallic materials is detected in situ and quantified ex-situ via scanning electron microscopy and confocal profilometry. With this knowledge, approaches to mitigate cavitation erosion or to even enhance the energy focusing are within reach.

## Highlights

• An intensified collapse by self-focusing of shockwaves emitted during the collapse is proposed as main mechanism of cavitation erosion of hard materials.
• Cavitation erosion occurs only for bubble collapses at close stand-off below γ≲0.2 where the self-focusing mechanism develops.
• Jetting mitigates cavitation erosion.
• Conditions to enhance the energy focusing and collapse intensity are presented.

## 1 Introduction

The ideal cavitation bubble is a spherical and empty bubble in an unbounded liquid that is exposed to a positive far field pressure. Such a bubble will collapse spherically and focus the liquid’s kinetic energy towards the bubble center [61]. Experimentally for millimeter-sized bubbles in water at atmospheric conditions, temperatures of thousands of Kelvin [21] and pressures of hundreds of atmospheres are attainable, with the remarkable conversion of mechanical energy into photons [10]. The ability of cavitation bubbles to focus energy leads to numerous applications, such as in recycling [52], [49], cell lysis and disinfection [17], peening of metals [81], [75], [77], eye surgery [90], and treatment of kidney stones [96]. A large part of the effects of cavitation stems from bubbles collapsing close to an interface, i.e. a solid or fluid boundary. Recently, the dynamics of a bubble at closest distance to a solid boundary have gained attention in experimental studies [98], [99], [67], [68]. Cavitation bubbles at interfaces in general develop non-spherical dynamics and can produce fast and microscopic flows. Here, their energy focusing ability leads to challenges, too. For example while in ultrasonic surface cleaning the wall shear stress from cavitation bubbles collapsing near the boundary is exploited [54], [65], the force could also damage delicate structures. The destructive potential of cavitation is also encountered in traumatic brain injury [25], [40] as well as in high speed turbomachinery. These challenges have attracted numerous research studies and advanced the field considerably [22], [37]. Despite these advances, the fundamental mechanism by which a non-spherical collapsing bubble focuses energy sufficiently to cause cavitation erosion has not been unraveled. Historically, the phenomenon of liquid jetting has attracted attention, where the simulations from the work of Plesset and Chapman [59] confirmed the experiments from Benjamin and Ellis [5] and Lauterborn [43], i.e. the non-spherical collapse creates robustly a liquid jet that impacts onto the boundary. A number of numerical and experimental works have associated the impact of this jet onto the substrate with erosion [38], [53], [5], [24], [59], [7], [82], [8], [18]. In particular the water hammer pressure upon impact was made responsible, yet its importance remained inconclusive as in some works no damage from the jet was reported [73], [33], damage at certain bubble to wall stand-offs [82] or potentially little jet damage at certain stand-offs only [58].

Cavitation erosion has also been attributed to the shockwaves emitted during collapse and their interaction with the boundary. In experiments, the pressures of the shockwave were measured with hydrophones and their fronts were imaged based on the density dependent refractive index of water [82], [88], [71], [72], [50], [79], [26]. Here, rather large and intermediate distances from the bubble to the wall were investigated.

Computational fluid dynamics offers a route to obtain the pressure loading of the boundary from the jet impact and collapse, and thus to relate it to the material properties such as its yield strength. Investigations at intermediate stand-offs from the bubble to the wall suggest that peak pressures from the jet impact and torus-shaped collapse on the boundary are of similar strength, while in many scenarios the pressure of the torus collapse exceeds that of the jet impact [36], [13], [91], [45], [84]. From the aforementioned works, we know that the loading is sufficient to expect damage if the bubble collapse is driven by a surrounding pressure significantly larger than the atmospheric pressure or at atmospheric pressure only where shockwaves from the bubble collapse would meet in perfect numerical axis symmetry [45]. In experimental studies however, we find severe erosion already for cavitation at atmospheric pressure but not on the axis of symmetry where the numerical shockwaves meet.

Here we reveal that instead of axisymmetric shock focusing or jet impact reported previously, it is the non-axisymmetric self-focusing of energy that results to erosion. Here already a single bubble collapse at atmospheric pressure is sufficient to erode even hard metallic surfaces. We therefore suggest, that the reported non-axisymmetric self-focusing mechanism is the main cause of cavitation erosion in general.

## 2 Materials and methods

We experimentally study the surface damage from the collapse of single, laser-induced cavitation bubbles by high speed imaging of the bubble dynamics and shadowgraphy of the shockwave fronts, complemented with measurements of acoustic transients. At the same position close to a solid, *N* identical bubbles are generated sequentially. The location of damage surface is detected in situ relative to the bubble dynamics and combined with ex-situ confocal as well as scanning electron microscopy (SEM).

**Fig. 1:**
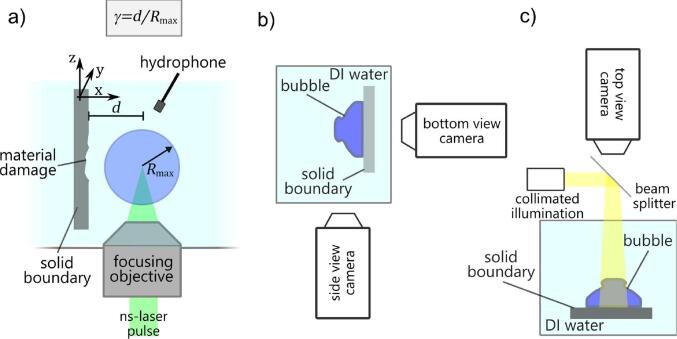
Experimental Setup. a) Side view on cuvette with definition of non-dimensional stand-off distance γ. b) Top view of cuvette – the bottom view camera can be moved to the left side for a top view of the bubble. c) Inline illumination with beam splitter for in situ imaging of damage.

Erosion tests are conducted in the experimental setup sketched in Fig. 1. A single bubble is generated in de-ionized water in a glass cuvette via optic cavitation with a pulsed laser (Litron nano S, dimensions of cuvette approximately 50 × 50 × 80 ${\mathrm{mm}}^{3}$, focusing objective: Mitutoyo 50×, numerical aperture NA = 0.42, nominal working distance: 20.5 mm, in–house modified with a water-tight sealing). The focusing objective is integrated into the cuvette bottom, i.e. laser and buoyancy point into the same direction. The bubble reaches a maximum diameter of about 1 mm and its dynamics is recorded in most cases in transmitted light with two high-speed cameras: Photron Fastcam Mini AX operated at 130,000 frames per second, and Shimadzu HPV-X2 operated at up to 5 million frames per second. Depending on the imaging scale required, the cameras are equipped with changing objectives: either 5×, 10× and 20× Mitutoyo Plan Apo microscope objectives (working distances 34 mm, 34 mm, 30.5 mm, respectively, and numerical apertures of 0.14, 0.28, 0.28, respectively), alternatively, a macro lens (Canon MP-E 65 mm) was used. To avoid laser beam absorption at the solid boundary, as this would produce spurious bubbles or direct material ablation, the laser is focused parallel to the boundary, implying some clipping of the laser beam. This however, does not affect proper bubble generation which we confirmed for a free bubble (i.e. without boundary) by covering half of the focusing objective aperture [67]. To correlate the location of the damage with the location of the bubble dynamics, the camera view can be varied as shown in Fig. 1c). The metal substrate and the bubble dynamics are imaged in top view of the substrate with an inline illumination using a beam splitter.

**Fig. 2:**
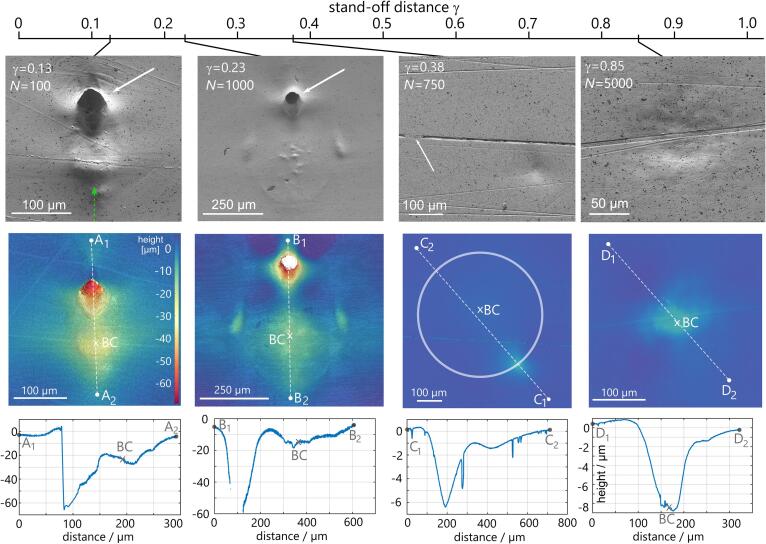
Damage morphology on single-crystal silver sample surface for increasing stand-offs γ=0.13,0.23,0.38, and 0.85, from left to right. The top row shows SEM images of the damage. γ denotes the stand-off, and *N* the number of cavitation bubbles created consecutively at the same spot. The green arrow in the first tile shows the direction of the incoming laser beam and buoyancy for all samples in the figure. In the second row the surface morphology is plotted color-coded, where the position of the projected bubble center is marked with “BC”. Height profiles along selected lines connecting the points $\overset{‾}{A_{1}\;A_{2}},\overset{‾}{B_{1}\;B_{2}}$ etc. are shown in the third row. Erosion is only produced for the two smaller stand-off distances.

The shockwaves emitted during the bubble collapse are visualized with shadowgraphy. We freeze the bubble dynamics and the shockwave propagation in time by illuminating the scene with an expanded laser pulse train (Ekspla Femtolux, wavelength 515 nm, pulse duration 220 fs) where each frame is illuminated with a single laser pulse. For convenience the laser light is brought to the experiment via a glass fibre. To record the acoustic emissions of the bubble, we employ in total three hydrophones of two types: two piezoelectric PVDF hydrophones that are rather sensitive type (Müller-Platte needle probe with rise time 10 ns and 10 MHz bandwidth, 0.6 mm tip radius) and one less sensitive yet higher-bandwidth fibre optical hydrophone (ONDA HFO690, spatial resolution 100μm, 100 MHz bandwidth, −3 dB, estimate rise time of 0.35/100MHz≈3.5ns). The measurement signal from the glass fibre hydrophone is deconvolved and converted to physical pressures following the manufacturers procedure. This requires a continuous monitoring of the water temperature which fluctuated by less than 1 °C, i.e. can be considered constant at 21 °C here. The distance from the hydrophones to the location of bubble collapse is about 5 mm to 8 mm for the needle hydrophones and about 2 mm for the fibre optic hydrophone. The hydrophones allow for a simple and reliable determination of the bubble life time $T_{L}$ (the time from plasma seeding to bubble collapse) as during both events a characteristic shockwave is emitted. The data is sampled with an oscilloscope (Teledyne LeCroy WavePro 404HD at 1 GHz sampling frequency with the internal hardware low-pass with 500 MHz cut-off frequency enabled). Close to the bubble, a solid sample is placed. Repeated impacts by single, almost identical bubbles result into damage of the sample, which is examined ex-situ with areal confocal profilometry (Mahr nanofocus μsurf custom P) and scanning electron microscopy (SEM, Thermo Fisher Scientific Quanta 650 FEG). The bubble stand-off distance to the boundary and the number of bubble collapses per location is varied, and for each combination of parameters a pristine position on the sample surface is chosen. From the surface profiles, eroded volumes, depth and areas are calculated using Matlab. We employ samples of glass to ease the imaging (see Fig. 1) and a number of metal samples for the erosion tests, i.e. pure silver, aluminium (5154 alloyed with magnesium), brass (63Cu37Zn), and stainless steel (V2A, 1.4301, rolled). The samples were cut as received into small plates with a size of 15×15×2 mm^3^ and polished by hand. Vickers hardness was measured for aluminium alloy, copper brass and the stainless steel as 65HV5, 72HV5 and 167HV5, respectively. The silver sample is a single crystal (MaTecK, purity: 99.999; orientation: (100), fcc, dimensions 15 mm × 6 mm × 1 mm, Young’s modulus 83 GPa, shear modulus 30 GPa, bulk modulus 104 GPa, manufacturer information). Owing to its single crystal structure, it has the advantage of exhibiting the same well-defined mechanical material parameters over its entire surface. The crystal was polished by hand with alumina polishing particles. The polishing procedure resulted in some polishing marks on the substrate which are visible for example in Fig. 2 as horizontal scratches. All scratches were sufficiently small to not alter the bubble dynamics, which was confirmed by comparing the bubble dynamics at locations with and without polishing marks. As a benefit, the marks helped to determine if at regions of a recessed surface level erosion took place or the underlying bulk material was only compressed. In addition, they facilitated the correlation of the bubble position with respect to the damage pattern in the top-view imaging series with the ex-situ profilometry. For this correlation we determined the coordinates of the (projected) bubble center in the high speed imaging taking the bubble centroid and measuring distances to characteristic surface features that are well-detectable both in- and ex-situ such as scratches or cavitation damage. We indicate the projected bubble center (“BC”) in the ex-situ surface analysis and estimate the optical magnification-dependent accuracy of the localization to be better than 10% of the geometric scale bar indicated in each respective image.

The bubble dynamics depends delicately on the stand-off. In particular in the small stand-off regime, already a few micrometers decide if the collapse dynamics result in the formation of the needle-jet [47], [67]. For precise control of this parameter the samples are translated with a motorized three-axis stage (x,y,z-direction, Physik Instrumente M-410.DG). We express the distance from the bubble to wall in non-dimensionalized form using the stand-off parameter γ. It is defined by $\gamma =d/R_{\max}$ where *d* is the distance of the bubble seeding center to the wall, and $R_{\max}$ the bubble radius at maximum expansion (see Fig. 1a). $R_{\max}$ is measured in direction perpendicular to the solid as discussed in [68]. To conduct a stand-off dependent study, a pristine surface areas of the sample is positioned close to the spot of bubble generation and a number *N* of identical bubbles are generated. Then the sample is translated to a yet untreated surface area nearby and the stand-off is varied. In a similar fashion studies where not the stand-off distance but the number *N* of bubbles collapses is varied are conducted. For the fibre optic hydrophone it is important to maintain a constant angle between the acoustic source (collapsing bubbles) and the fiber because of its directive sensitivity. Therefore the optic hydrophone is moved together with the sample. A second important detail is the waiting time between successive bubble generations, thus to allow gaseous fragments from one laser-generated bubble to move away and/or dissolve such that they do not interfere with the dynamics of the following bubble. Here we chose delays longer than Δt=8 s as we found in preparatory tests that above this delay the damage pattern converged, i.e. it did not vary measurably with increased delay times, and the high speed imaging confirmed that the bubble dynamics were unaffected from potentially remaining gas fragments. For the experiments reported, more than 20 000 single bubbles were generated. For the erosion measurements, the mean bubble radius for the silver sample was ${\overset{‾}{R}}_{\max}\approx 540\;\mu m$ and for all other materials the mean maximum bubble radius was around ${\overset{‾}{R}}_{\max}\approx 700\;\mu m$ (standard deviation for the same material smaller than 50μm). To aid comparison of the pressures, we scale the recorded pressure $p_{\mathrm{rec}}$ by $p=p_{\mathrm{rec}}{\left(\frac{{\overset{‾}{R}}_{\max}}{R_{\max}}\right)}^{1.5}$ because the bubble energy *E* scales with $R_{\max}^{3}$ and the acoustic pressure, in general, with $\sqrt{E}$. The distance of the hydrophone to the spot of bubble collapse $d_{h}$ is calculated from the propagation delay of the shockwave from bubble generation at γ=0, i.e. when the plasma is generated directly at the solid, using a constant speed of sound in water at 21 °C as $c_{0}=1483\;m/s$. For a direct comparison of the pressures measured at different distances, pressures are normalised to a “standard distance” for which we chose 700μm which is approximately the mean maximum bubble radius. For this normalization we consider viscous absorption by the following scaling: $p=p_{\mathrm{rec}}\frac{d_{h}}{{\overset{‾}{R}}_{\max}}\exp\left(-\alpha_{v}({\overset{‾}{R}}_{\max}-d_{h})\right)$, where $p_{\mathrm{rec}}$ is the pressure measured at the hydrophone position and $\alpha_{v}$ the viscous absorption coefficient [6]. It is calculated by $\alpha_{v}=\frac{2\mu \pi^{2}f^{2}}{\rho_{0}c_{0}^{3}}(\frac{4}{3}+\frac{\mu_{b}}{\mu })$ with $\mu =1\;\mathrm{mPa}\;s,\mu_{b}=3.1\;\mathrm{mPa}\;s$, and $\rho =1000\;\mathrm{kg}/m^{3}$. To estimate the characteristic frequency *f*, we take the Nyquist frequency of the rise time of the pressure transients measured with the high bandwidth fibre optical hydrophone, which is about $f=1/50\;{\mathrm{ns}}^{-1}$ (see Fig. 8c). Combining both equations yields:

(1) $$p=p_{\mathrm{rec}}{\left(\frac{{\overset{‾}{R}}_{\max}}{R_{\max}}\right)}^{1.5}\frac{d_{h}}{{\overset{‾}{R}}_{\max}}\exp\left(-\alpha_{v}({\overset{‾}{R}}_{\max}-d_{h})\right),$$

which is used to scale all pressures presented here.

## 3 Results

### 3.1 Surface damage

*Damage Morphology* The damage on a silver single-crystal surface as function of the stand-off distance γ is shown in Fig. 2.

When the bubble is generated very close to the solid surface (γ=0.13), severe erosion is produced. A remarkably confined, almost drilled-like hole is visible (see arrow) that forms within N=100 successive impacts of nearly identical bubbles. The opening diameter is about 30μm and the depth 102μm (aspect ratio: 3.4). It is inclined by a small angle towards the surface normal. Consequently, there is some undercut and the maximum depth was measured with the sample tilted suitably in the confocal microscope.

Increasing the distance to γ=0.23, a very similar hole is eroded yet it now needs about ten times more bubble impacts. Thus a small change in distance reduces the errosivness of the cavitation bubble collapse considerably. Besides, in the center several small indents are seen, and a ring onto which the bubble collapses is faintly visible. The hole is too deep to be resolved in the single confocal image resulting in some masked data points (shown in white).

Only a few tens of micrometer further away from the boundary, at γ=0.38, after a similar number of N=750 bubble collapses no erosion is detected. At the bubble center (BC) we find only a minor indent with a depth of about 1.5μm and at some distance from BC there is a somewhat bolder indentation. The latter has a depth of ≈6μm and coincides with the extent of the second collapse after rebound, which is indicated with the circle. The more detailed analysis of the bubble dynamics (see below) will reveal that this damage is indeed the result of the second bubble collapse. These indendations are purely compressive, i.e. are not connected with erosion. This can be seen by the intact surface structure, the remaining polishing particles (dark grains) and the polishing marks that still pass along the indented surface. Within the depicted ring another detail can be observed: the indentations from polishing are filled, which we explain with shear induced transport of material (see arrow in the SEM image). Besides the silvers general ductility, the single crystal configuration here facilitates shearing of atomic layers.

For the largest stand-off γ=0.85±0.05, the surface has a deepening of about 9μm speckled with several smaller indents. The maximum depth is located near the projected bubble center (“BC”). Here again, no erosion has taken place, even after 5000 bubble collapses.

Comparing all images, only at the smallest stand-off, there is also some damage from bubble seeding seen by the indentation around “BC” of about 50 to 100μm in diameter but without erosion. This plastic compression can be attributed to the blast wave that follows the laser-induced optical breakdown during bubble seeding, as shape, size and position of indentation and plasma match neatly, see Appendix B. In Appendix A we further study the evolution of the damage with increasing *N* and the effect of different materials. The latter reveals that the same hole structure is also found for harder and for more brittle materials, demonstrating that surface erosion is basically unchanged with material, Appendix C. This suggests that the same cavitation erosion mechanism occurs independently of the metal material.

**Fig. 3:**
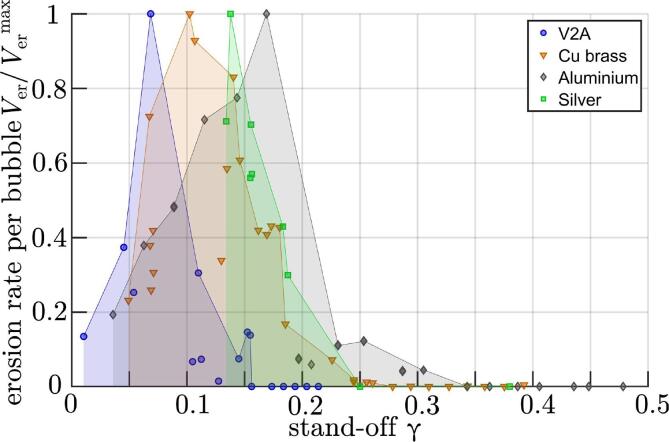
Erosion rate: volumetric material loss rate per bubble as function of the stand-off for four different materials. Each curve is normalized to its respective maximum.

*Erosiveness of Collapse – Volumetric Material Erosion Rate.* The severe erosion occurs in the shape of a drilled like hole. The erosion rate associated with it is presented in Fig. 3 for four different materials. We express the erosion rate as volume loss rate per cavitation bubble where the erosion volume is measured with confocal areal profilometry. We use the term “erosiveness” when comparing the material loss rate per bubble. It is a lower boundary as the optical profilometry does not resolve undercuts which are present for some of the deepest erosion holes. For better readability, each curve is normalized to its respective maximum and local maxima are connected to a curve. The respective maximum erosion rates per bubble collapse are $V_{\mathrm{er},\max}^{V2A}=472\;\mu m^{3},V_{\mathrm{er},\max}^{\mathrm{brass}}=869\;\mu m^{3},V_{\mathrm{er},\max}^{\mathrm{alu}}=2877\;\mu m^{3}$, and $V_{\mathrm{er},\max}^{\mathrm{Ag}}=1938\;\mu m^{3}$. For the more resistant sample V2A, N=75 was chosen, for brass and aluminium alloys N=20, and in the case of silver *N* was either 10 or 100. The standard deviation of γ for the repeated impacts at each position are 0.005,0.003,0.008 and 0.011, respectively. It can be seen that for stand-off distances above a γ-threshold the erosion rate is negligible, it is even identically zero for the number of bubbles studied, i.e. the stand-off can be divided into an erosive and a non-erosive regime. Measurements not shown were carried out up to γ=1.5 and again did not result in erosion during the first bubble collapse.

Furthemore, the data suggests that for the softer materials the erosion rate is larger and the erosive stand-off range is a little more extended, the erosion threshold for the aluminium alloy is γ≈0.25, and γ≈0.15 for the stainless steel. Remarkably, even on the steel sample at stand-offs around the erosion rate maximum, already after only one bubble collapse, visible damage is produced. In addition the data indicates that for γ→0 the erosion rate may decrease again, thus there exists a most erosive stand-off.

### 3.2 Bubble dynamics and shadowgraphy of shockwave emissions

First, we analyse the bubble dynamics in the erosive stand-off regime. Then we compare it to bubble dynamics at a somewhat larger stand-off to identify the specific differences that make the latter non-erosive.

#### 3.2.1 Bubble dynamics in the erosive regime

**Fig. 4:**
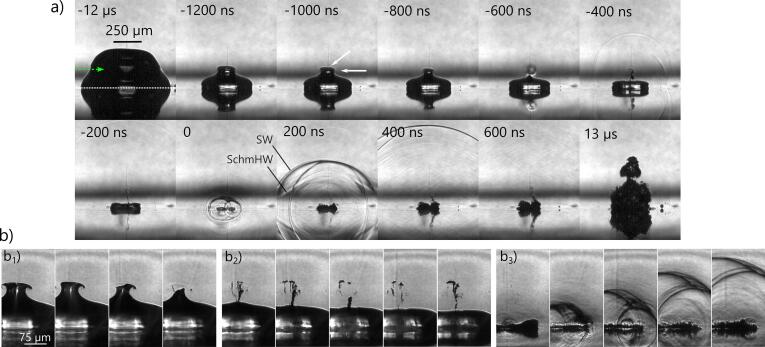
Bubble shape dynamics and shockwave emissions at small stand-off in the erosive regime. *a)* Side view: Stand-off is γ=0.06. The boundary is indicated by the thin dashed line in the first frame, and the solid surface at the bottom shows a mirror image of the bubble. The arrow in the first tile indicates the laser beam and buoyancy direction. Times are indicated in each frame with respect to the collapse frame. The interframe time is 200ns, except for the first and the last frame. Prominent features are the needle-jet formation and the strong collapse shockwave emissions seen at t=200 ns with spherical wave fronts (SW) and conical fronts from Schmidt head waves (SchmHW). *b)* Close-up of key features in side view: *b*_*1*_*)* cap collapse, *b*_*2*_*)* needle-jet piercing through the bubble and impact, *b*_*3*_*)* collapse shockwave emissions. To virtually reduce the interframe time, these frames are taken from five repeated recordings of identical bubbles at 5 MHz and aligned in time according to their respective instance of collapse which is derived from the position of shock fronts. The interframe time can then be estimated as 200ns/5=40ns.

A high-speed imaging series of the bubble dynamics in the erosive regime is presented in Fig. 4a. In this regime the bubble expands so close to the boundary that it obtains in first approximation a hemispherical shape at maximum expansion (not shown). Yet, later during collapse the shape changes. At time t=-12μs prior to the collapse, which is 135μs after seeding ($R_{\max}=787\;\mu$m) the bubble reveals a circumferential kink (white arrow) and a cap (black arrow), see t=-1000 ns. These two features are the result of a flow parallel to the boundary that occurs only for small stand-off distances. A movie of this dynamics is available, see Movie S1. Between t=-600ns and t=-400ns the boundary-parallel flow closes circumferentially on the symmetry axis and thereby pinches off the collapsing bubble cap. This process results in the shockwave visible at t=-400ns, in Fig. 4a. This shockwave in dependence of the stand-off is further studied in Appendix F. The convergent flow and collapse of the cap drive a needle jet through the bubble towards the solid. The needle jet is only a few micrometers in diameter, and previously we have measured averaged velocities of $\approx 1000\;{\mathrm{ms}}^{-1}$ [67] while peak velocities are even faster. Detailed simulations are available in Lechner et al. [46], [47].

The needle jet is visible inside the bubble at t=-400 ns where it already has impacted onto the solid and created a fine spray in the bubble interior. The minimum bubble volume is reached around t=0 resulting in much stronger shockwave emission. From the radius of the shockwave front the time of collapse can be estimated to have happened at t≈-20ns.

In the successive frames in Fig. 4a, 200 ns⩽t⩽600 ns, the shockwaves are propagating away and tend to form one spherical front after some distance. Yet at time t=0 two prominent separated shockwaves are shown that were emitted at two different times and from two different locations. Besides the circular fronts, straight linear fronts are seen left and right of the bubble at t=200 ns. These belong to conical Schmidt head waves originating from bulk waves and surface waves in the solid [86], [95]. They are discussed in Appendix E.

The last frame in Fig. 4a, t=13μs reveals the bubble at some later stage during its rebound. This shape is rather different from the shape of the rebounding bubble for larger stand-offs. There the bubble is elongated parallel to the boundary while here it is normal to the boundary. The key events are detailed in magnified sequences with an increased temporal resolution in Fig. 4b. Fig. 4b is split into b_1_) the cap collapse, corresponding to t=-800 and -600 ns in Fig. 4a, b_2_) the needle-jet piercing with the splashing after impact on the solid (t=-600 to -400 ns). In the second frame of Fig. 4b_2_) the needle jet is captured during its passage through the vapour phase revealing a fragmented tip. Possible scenarios causing this atomization could be a destabilization by the hypersonic needle-jet speed [20] or by the shockwave emitted upon cap collapse and its reflections. The needle-jet tip-velocity can be roughly estimated from frame 2 and 3 of b_2_. Its propagation distance is estimated as 60μm and the approximate interframe time is 25 ns, suggesting a needle-jet speed as large as $v\approx 2400\;{\mathrm{ms}}^{-1}$.

**Fig. 5:**
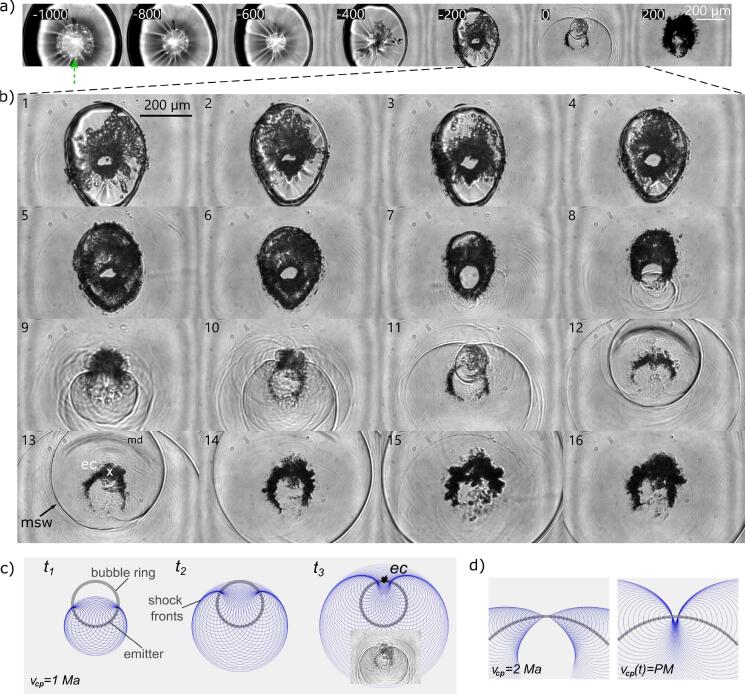
Bubble dynamics and shockwave self-focusing in erosive regime. For the corresponding video please see Movie S2. *a)* High-speed recording with times stated in nanoseconds with respect to the collapse frame t=0, similar as in Fig. 4 but seen here through a glass substrate at γ=0.12. *b)* Further frames in between the indicated frames from nine subsequently generated bubbles, temporally aligned as above. The interframe time can be estimated as 200ns/9=22.2 ns, thus, about 333 ns of the bubble dynamics are covered. The arrows indicate the main shockwave (“msw”) and its epicentre (“ec”), which is also the location of cavitation erosion. The temporary crescent stripes stem from some internal material fracture damage (“md”). *c)* Schematics of collapse intensification through self focusing by shockwave emission from a sonic ring collapse, i.e. from a collapse that proceeds along the ring from bottom to top at $v_{\mathrm{cp}}=1$ Mach. The emitters along the ring are indicated by dots, wave fronts by the circles for subsequent times $t_{1}$ - $t_{3}$. The wave fronts converge towards the erosion center (“ec”). The inset in the last image shows a comparison to the experimental shock geometry from b). *d)* Schematics of shockwave convergence shortly before complete ring collapse for $v_{\mathrm{cp}}=2$ Ma, and the perfectly matched ring-collapse velocity according to Eq. 2 yielding an ideal spatio-temporal convergence of all wave fronts into one point.

Before discussing the events leading to the strong shockwave formation in Fig. 4b_3_), we study the collapse from the top view, i.e. through a transparent glass sample, sketched in Fig. 1b. Fig. 5a starts at t=-1000 ns where the neck is closing resulting in a pinch off of the cap, which collapses at t=-400 ns ($R_{\max}=629\;\mu m,T_{L}=121.7\;\mu s$).

Consequently, bubble fragments on top of the bubble at the former cap position appear (compare to the side view in Fig. 4). At -200 ns the bubble is already pierced and the jet has impacted on the solid. This can be deduced from the clear view through the bubble center, indicating the absence of a liquid–gas interface. The frame at t=0 captures the bubble right after reaching minimum volume. We would like to stress the high velocity by which the phase boundaries collide. This velocity can be estimated from the frames at t=-200 ns and t=0, and the distance the main radial shockwave has travelled at t=0 to obtain the instance of shockwave emission. To account for the speed of finite amplitude wave we use the measurement data from Geng et al. [23] (Fig. 5 in that work) who measured the propagation distance of the shockwave from plasma seeding as a function of time. From this analysis in similar time series we obtain (averaged) bubble wall velocities series above 1000 m/s, implying double the values for the collision of the inner and outer torus wall as lower boundary.

The frame at t=200 ns shows the initial rebound stage. The entire ring rebounds with a complex interface morphology, likely caused by the interaction of the shockwave with the interface, i.e. Richtmyer-Meshkov [11] instabilities, splitting of the torus, and the simultaneous re-expansion of many gas fragments.

The origin of the emission of the two shock waves seen at t=0 is studied in Fig. 5b at increased temporal resolution from nine imaging series. The first two rows show the needle-jet impact and splashing while the torus is collapsing. At frame 8 the ring has collapsed in the lower region emitting a shock wave. At frame 11 also the last gas volume has collapsed. Thus, both views (Fig. 4b_3_ and Fig. 5b) at this high temporal resolution reveal that the collapse progresses along the ring - in side view in the direction left to right, in top view in the direction bottom to top. During this collapse propagation along the ring, shockwaves are emitted and superimpose constructively. We observe that the collapse of the last remaining part of the gas phase proceeds particularly violent resulting in the sharpest shock front. Simultaneous observation of the bubble dynamics and the surface damage, reveals that at this position of strongest shockwave emission the prominent erosion in form of the drilled like hole is produced, see Appendix G. The shockwaves emitted along the collapsing bubble ring superimpose and converge towards the bubble fragment that collapses last. There, they consequently drive an intensified collapse. A rough estimate of the effect can be obtained by calculating a characteristic velocity $v_{\mathrm{char}}$ of the bubble wall from the Rayleigh collapse time τ [61]. Taking $v_{\mathrm{char}}=R_{\max}/\tau$ one obtains $v_{\mathrm{char}}\propto \sqrt{\Delta p}$ where Δp can be considered the pressure difference between bubble interior and the liquid (at infinity), which is usually 1 atm, but due to the shockwave, orders of magnitude larger. Thus, the collapsing torus accelerates and intensifies the collapse for the last remaining bubble fragment of the ring. Fig. 5c presents a simple model for shockwave propagation with emission sites located on a ring and viewed from the top. We assume equally spaced emitters indicated with a dot, each emitting a circular single wave front that propagates with the speed *c*. The collapse and therefore first shockwave emission starts from the lowermost point on the ring, which is approximated with a circle. We introduce the collapse propagation velocity $v_{\mathrm{cp}}$ such that the topmost point of the ring collapses after a time $\pi r/v_{\mathrm{cp}}$, where πr is half the ring perimeter. In Fig. 5c we set $v_{\mathrm{cp}}=c=1$ Ma and depict the shock fronts for times $t_{1}<t_{2}<t_{3}$. As a result the wave fronts form a cardioid and converge to the erosion center “ec” at $t_{3}$. The density of lines is related to the pressure, thus one expects a considerably higher pressure at the top via a caustic. The inset for $t_{3}$ shows that the simple model resembles the instantaneous image of the shockwaves in the experiments. The reason is that in the experiment the strength of the shockwaves is related to the contrast, similar to the graphical front representation.

This shockwave focusing requires an asymmetry, i.e. the ring must collapse at one location first. Fig. 5b suggests that the collapse proceeds with a velocity of more than $2500\;{\mathrm{ms}}^{-1}$ along the ring, and the time interval $T_{\mathrm{cp}}$ between collapse of first and last ring segment is only ≈66 ns, i.e. less than 0.6 per mille of the bubble life time. We can thus expect that already minute asymmetries in the experiment could result to this condition. Three potential sources of asymmetry can be identified in our setup: buoyancy, seeding plasma asymmetries, and the finite extension of the solid surface.

The direction of collapse coincides with buoyancy in our setup, i.e. the top in the image corresponds to the upper part in the physical setup. To estimate the effect of buoyancy in bubble dynamics the buoyancy parameter is frequently used [93]. It can be considered the inverse of the bubble Froude number: $\sqrt{\rho g{\overset{‾}{R}}_{\max}/(\Delta p)}=0.0085$, where Δp≈1 bar. Thus, even though from this estimation buoyancy seems negligible for the overall bubble dynamics, the buoyancy parameter is of the same order of magnitude as $T_{\mathrm{cp}}/T_{L}$ and it may be decisive in the introduction of small asymmetries. A conical or ellipsoidal shape of the seeding plasma is common and results from the altered absorption and reflection properties of the plasma during the laser pulse which makes the plasma grow towards the laser [89]. This effect, while present here, was minimised by reducing spherical aberration with beam limiting apertures and a high-NA focusing objective. The laser pulse was focused from bottom to top in Fig. 5 and was coinciding with the direction of collapse propagation in all cases of the first collapse. Additional effects may stem from the finite extension of the solid surface. While for decreasing small stand-offs this effect should decrease as well, still it may introduce asymmetric flows.

In Fig. 5b also some material damage in the glass sample can be detected as a dark and curved structure labeled “md”. This structure is only visible for some tens of nanoseconds, approximately the time during which the shockwave travels across it, and after some bubble collapses at the same spot. As we do not detect roughening or erosion of the surface, the material is likely fractured within and these internal fracture planes opens only under dynamic loading of the material. Leaky Rayleigh waves have been reported to induce fracture damage on glass samples [95] and may also play a role here.

**Fig. 6:**
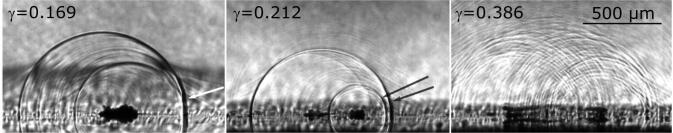
Comparison of collapse shock front configurations in side view in the erosive regime (left), the transition regime with milder erosion (center) and the non-erosive regime (right). Images taken on an aluminium sample, laser incident from the left.

*Stand-off Dependence of Shockwave Self-Focusing.* After establishing that the focusing of the shockwaves is related to the erosion, we are now using the side view to look at the effect of the stand-off distance on the shockwave emission. Fig. 6 depicts the collapse shockwave emission for three stand-offs increasing from left to right. For the smallest and most erosive distance γ=0.169 two prominent spherical shock fronts and a number of weaker spherical fronts are visible. For the two prominent spherical waves, the one with the larger diameter results from a superposition of many local emissions along the collapsing torus. The smaller spherical wave originates from the site of the intensified collapse and cavitation erosion. Interestingly, the two spherical shock fronts coincide on the right side as indicated with the arrow in Fig. 6, γ=0.169. Thus the wave fronts propagating to the right in Fig. 6 are superimposing constructively implying an amplification of the shock front. Additionally, we find head waves at two angles of approximately $15^{\circ }$ and $29^{\circ }$, which would correspond to lateral wave velocities in the solid of 5600 m/s and 3100 m/s, respectively, which is of the expected magnitude (see Appendix E).

The second stand-off presented in Fig. 6 has a slightly larger stand-off γ=0.212 that is at the threshold where erosion occurs, see Fig. 3. Still, two main spherical shock fronts with their centers at the left and right side of the torus remain, yet the contrast of the shockwaves has reduced. Also the larger spherical wave front is advancing the smaller spherical front by approximately 50μm. Both fronts are indicated by arrows.

By increasing the distance only slightly further to γ=0.386 we are leaving the erosive regime. Then instead of two pronounced shockwaves many isolated spherical shock fronts appear. These have a considerably smaller contrast. We explain this lower contrast with a missing constructive superposition of the wave fronts, i.e. the parts of the toroidal ring collapse individually without focusing the acoustic energy to its right part.

Thus, within the erosive regime the efficiency of constructive interference of collapse intensification gradually changes in accordance to the gradual changes of the erosion rate.

*Collapse Propagation Velocity and Energy Focusing.* Shock focusing can only occur when $v_{\mathrm{cp}}$ is sufficiently large, i.e. similar to the shockwave propagation velocity, for which we assume *c*. Fig. 5d shows magnified views of the upper locus “ec” of the ring. The left tile plots the shock fronts for $v_{\mathrm{cp}}=2$ Ma, and we find that the collapse velocity must approach the speed of sound to form a caustic region. If the ring would collapse simultaneously, i.e. $v_{\mathrm{cp}}\to \infty$, the shock fronts would constructively superimpose at the center of the ring [87], [70].

What would be the optimum velocity $v_{\mathrm{cp}}$ to generate maximum amplification? To answer this question we make a number of simplifying approximations, namely we assume a constant speed of propagation *c*, neglect Mach stems effects, i.e. interference of head waves along the solid surface with the shockwaves in water, and only consider the upper part of the ring. We assume that the collapse velocity is perfectly matched once all wave fronts generated by the collapse reach the erosion center at the same time. It follows from geometric considerations that account for the relation between the chord length and the circle segment from each emitter along the ring to the erosion center, that the collapse velocity is perfectly matched when: $\omega (t)=2\mathrm{asin}(\mathrm{ct}/(2R_{\mathrm{rc}}))$, where ω is the angular collapse velocity and $R_{\mathrm{rc}}$ the collapse ring radius. Integration yields a time-dependent collapse velocity along the half-ring for maximum energy focusing as:

(2) $$v_{\mathrm{rc}}(t)=c\frac{1}{\sqrt{1-c^{2}\tau^{2}/\left(4R_{\text{rc}}^{2}\right)}}\text{,}$$

where $\tau \left(t\right)=\sqrt{2}R_{\text{rc}}/c-t$, and *t* = 0 denotes the beginning of the half-ring collapse. The resulting shock fronts for this condition are shown in Fig. 5d.

#### 3.2.2 Bubble dynamics in the non-erosive regime

**Fig. 7:**
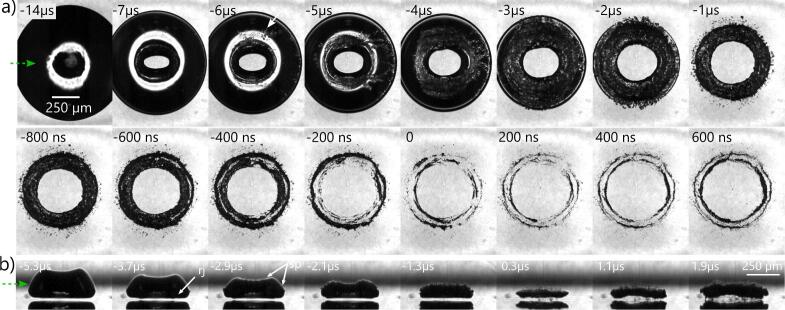
Shape dynamics during collapse at intermediate stand-off in the non-erosive regime. *a)* Bottom view through a glass substrate ($\gamma =0.571,R_{\max}\approx 620\;\mu m,T_{L}=135.26\;\mu s$). The top row shows the comparably slow impact of the regular jet (velocity 50 - $100\;{\mathrm{ms}}^{-1}$) and subsequent splashing with fragmentation of the main bubble into small micrometer-sized bubbles and more than one ring onto which the bubble collapses. Shockwave emission is delocalized, asynchronous, and with small amplitudes (fronts are only faintly visible, see t=0 to 200 ns). *b)* Side view with similar dynamics at γ=0.816. “rj” denotes the protrusion from the piercing of the regular-jet, and “sp” splash impacts. Videos of the dynamics are provided in the Movie S3.

Next we want to identify how slight changes in the stand-off distance result to non-erosive bubble collapses without the energy focusing mechanism. In this regime a regular jet instead of the needle jet threads the bubble and shapes it into a larger torus, see Fig. 7. At t=-7μs in Fig. 7a the regular jet impacts onto the substrate as revealed by the clear view through the center. Upon impact the jet is reflected as a sheet under some angle towards the surface normal. Tong et al. [83] found this flow in axisymmetric simulations and termed it as splashing. In experiments the flow is three dimensional and more complex: the splash sheet becomes unstable. It pinches off droplets from its rim that collide into the still shrinking bubble interface, see arrow at t=-6μs in Fig. 7a. Upon impact of these droplets, gas and vapour become entrained into the liquid resulting in a complex phase boundary, see t⩾-2μs. Because the jet splashes upwards and away from the glass sample, it also splits the toroidal bubble into two rings. Around t=-200 ns the inner ring collapses and shortly afterwards the outer ring collapses until about t=200 ns. Thereby many rather weak shockwaves are emitted that originate from the center lines of the rings where individual bubble fragments collapse.

Fig. 7b shows the dynamics in side view, for which a somewhat larger stand-off was chosen, as it allows for a better visualization of the regular-jet impact, yet the key features can be considered the same as in Fig. 7a with slight differences in the timing. The torus radius of the jet can be estimated from the protrusion indicated with “rj”. Here, the first shockwave emission can be seen already at t=-1.3μs before collapse, pointing to an extended collapse period. Emissions originate from several loci and show dim fronts, indicating low amplitudes. Interestingly, the collapsing torus is not in contact with the solid surface but is separated by a cushioning liquid layer, which we previously measured to be 16μm thick for this stand-off [63]. While this collapse does not cause damaging, the second collapse of this bubble after its rebound can result to rather weak but noticeable damage, in form of an indentation after a sufficient number *N*. The mechanism involves again similar energy focusing during the second toroidal collapse which is studied in Appendix H. Further effects of jetting on erosion and destabilization of the gas liquid interface is described as a function of the stand-off distance in Appendix I.

**Fig. I.19:**
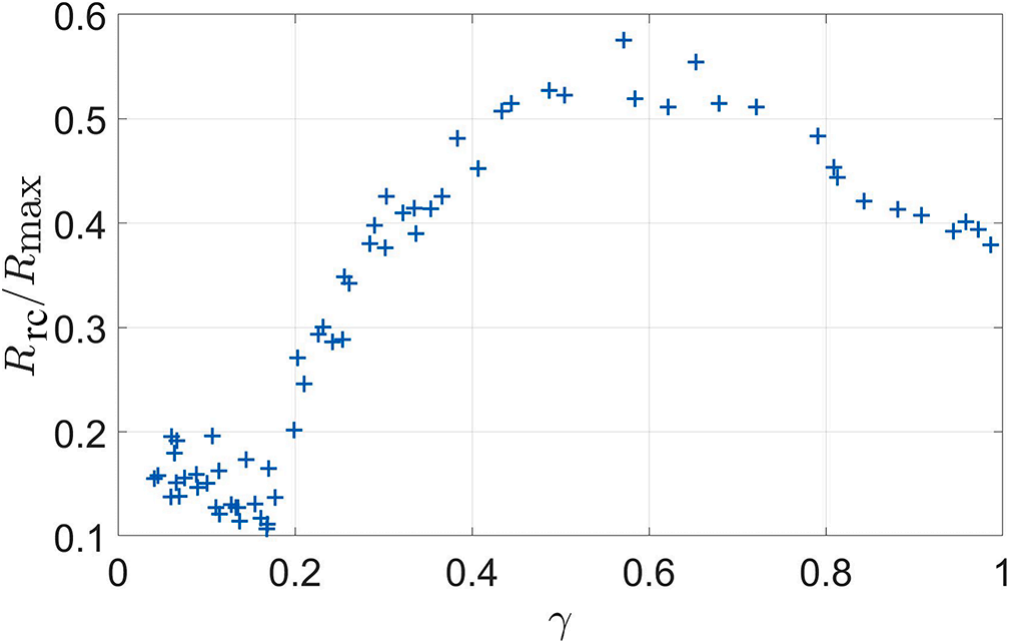
Radius of the ring collapse at the substrate as function of stand-off distance. Data is normalized on the respective maximum bubble radii: $R_{\mathrm{rc}}/R_{\max}$.

*Role of the Needle Jet in Cavitation Erosion.* As the stand-off goes to zero, the bubble dynamics becomes approximately that of a hemispherical bubble, which in potential flow approximation can be considered equivalent a spherical bubble due to symmetry considerations [68]. Consequently, for γ→0 a maximum energy concentration is expected. Jetting however hinders the energy concentration by splashing into the bubble interior and forcing the bubble into a ring collapse rather than a point collapse as detailed in 3.2.2 and Appendix I. The needle jet due to its competition with the regular jet increases the energy concentration during collapse as it results in a smaller collapse ring radius. Consequently, the needle jet and the erosive regime largely overlap. The needle jet itself however does not produce erosion despite its supersonic speed. This fact is supported through two observations: The needle-jet impacts onto the region of the bubble center (projected onto the solid) and not onto the erosion site, see for Example G.15c. There, the erosion site is located ≈60μm to ≈200μm away from the needle-jet impact region, i.e. on the ring of the collapsed bubble. At fixed $R_{\max}$, it is the further away the larger γ as the ring radius increases with the stand-off, see Fig. I.19. Secondly, the drilled-like hole is also produced in a regime where the needle jet is not formed. This is the case for example in Fig. 3.1 at γ=0.23.

Possible reasons for the absence of erosion from the needle jet are its rather small momentum and the apparent instability. However, the needle jet may be able to pierce through foils or softer materials.

### 3.3 Acoustic transients emitted during bubble collapse

**Fig. 8:**
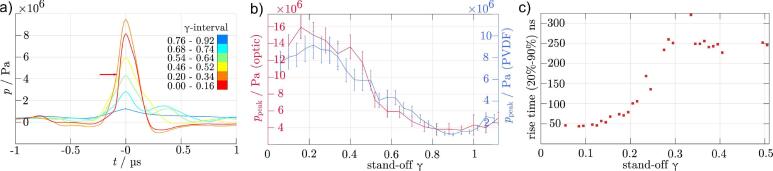
Acoustic transients from bubble collapse. Data is normalized on 700μm probing distance. *a)* Collapse pressure signatures for several representative γ-intervals at the aluminium sample taken with the PVDF hydrophone. The interval corresponding to the erosive regime (red curve) features a short rise time (horizontal arrow at rising edge) but not the largest peak amplitude. Curves are aligned to their respective maximum which can be related to the instance of collapse (t=0). *b)* Collapse peak pressures as function of stand-off distance. *Left axis*: Recorded with the optic hydrophone, (120 MHz bandwidth). *Right axis:* Recorded with the PVDF hydrophone (10 MHz bandwidth). Error bars give the standard deviation within each stand-off interval. *c)* Rise time (20% to 90%) of the collapse peak as function of the stand-off (120 MHz bandwidth).

The previous section revealed that there exists a critical threshold below which cavitation becomes erosive. Here we complement the imaging of shockwaves with acoustic measurements and aim to reveal acoustic patterns that distinguish erosive and non-erosive cavitation. Fig. 8a displays the acoustic transients emitted during the bubble collapse. The curves are averages over several measurements taken within the stand-off intervals indicated in the legend. These intervals are chosen such that similarly-shaped curves become aggregated. The acoustic traces are highly repeatable and do not change with progression of surface damage, see Appendix D. This in turn also demonstrates the repeatability of bubble seeding and shows that the dynamics can be considered to be unaffected by the progression of the surface damage here.

The central peaks in the figure result from the shockwaves emitted during the main collapse of the vapor cavity. For increasing stand-off distances, the peaks tend to broaden and the peak pressure decreases. While we generally find that the amplitude of the pressure increases with decreasing distance, the highest pressure does not occur in the most erosive regime (red curve, γ=0.00-0.16) but in the transition region between erosive and non-erosive stand-offs (orange curve, γ=0.20-0.34).

The peak pressures as a function of the stand-off are plotted in Fig. 8b using the fiber optic hydrophone (high bandwidth, low sensitivity, distance from bubble 1.9 mm) and the PVDF hydrophone (higher sensitivity, distance 4.9 mm). We scale both measurements to a common distance of 700μm as described in the Materials and Methods Section. More than 3000 bubbles with $R_{\max}\approx 720\;\mu m\;\pm \;55\;\mu$m were evaluated, and as no significant effect of the boundary material on the peak pressure could be noted, bubbles of different boundary materials were evaluated together. Both hydrophones show very similar values in magnitude and in trend. However, one would expect a more accurate pressure reading from the PVDF hydrophone for smaller peak pressures, i.e. for γ≳0.4 due to its better signal to noise ratio, and a more accurate reading from the optic hydrophone for the high pressure peaks at γ≲0.4 due to its higher bandwidth and closer proximity to the bubble, thus being lesser affected by dissipative and dispersive effects of wave propagation.

Previous measurements using single laser induced bubbles found a minimum at γ=0.9 [88], [53], [82], [26] that was also confirmed for single bubbles induced by focused ultrasound [12]. Here, in addition we provide measurements in the erosive regime for γ→0, where somewhat surprisingly, the pressure peak amplitude does not increase further but levels off or even decreases for γ<0.2. The peak pressure measured at some distance from the collapse site is therefore not a suitable measure for the erosiveness of the bubble collapse.

Next, we investigate the rise time of the pressure signal as a function of the stand-off in Fig. 8c, using the high-bandwidth optic hydrophone. Noise is reduced by averaging 250 p(t)-curves in γ-intervals of 0.01 for the glass sample with $R_{\max}=548\;\mu m\;\pm 35\;\mu$m. Here it is suitable to measure the rise time from 20% to 90 % to avoid cross-talk from the Schmidt head waves. In the erosive regime, the rise time is shortest and quickly increases once non-erosive stand-offs are approached. For γ>0.3, a plateau of about 250 ns is obtained.

The rapid increase of the rise time around γ≈0.25 correlates well with the reduction in erosiveness of the collapse. Thus, the erosive regime comes with a steep slope of the pressure wave front. The causes are the synchronized shock emission from the erosive ring collapse and the geometrically confined emission center of the main shockwave from the intensified collapse.

In Appendix E we address the effect of the boundary material on the acoustic emission during bubble collapse. It is shown that the central peaks remain unchanged, thus the collapse dynamics can be considered to be essentially independent of the material. Yet the Schmidt head wave precursors carry a signature of the material.

## 4 Discussion

Cavitation erosion of the metallic samples occurs at the final collapse of the progressive ring collapse by an energy focusing mechanism from emitted shockwaves only for distances of γ≲0.2. The collapsing torus emits constructively superimposing shockwaves. At the location where both fronts meet they force the remaining vapour phase, which is already in a late stage of shrinkage, to a *self-intensified* collapse with strong shockwave and head wave emission. It is this intensified collapse of the last part of the torus, which is responsible for the material erosion.

The intensified bubble collapse by an *external* shockwave has been reported by Sankin et al. [69]. Shockwave *induced* collapses, i.e. when a shockwave hits an essentially static bubble, are known to also cause violent bubble collapses [57], [55], [35], [34], [29], [42].

A shockwave can also be amplified through the collective dynamics of bubbles. Already simple geometric configurations where a shockwave is impacting on small number of bubbles in bubble arrays yield large energy concentrations [16].

Numerically, the shockwave induced collapse of an array of cylindrical bubbles [41], and groups of spherical bubbles [4] were studied, and an increase of the collapse pressures by a chain mechanism was observed.

For the tailored nanoparticle production by laser ablation of solids in liquid environments (LAL) a laser pulse is focused directly onto a submerged substrate and the material is ablated through plasma generation [3]. Alongside this process a laser-induced cavitation bubble is generated in the stand-off regime that is identified here as erosive. Bubbles in LAL show essentially the same dynamics as the single bubbles in the present work, in particular the circumferential kink in Fig. 4 that leads to the needle jet formation presented in [32], [51]. While the direct laser-material interaction clearly causes material ablation as the expanding bubble already contains some particles [1], [62], the influence of the cavitation bubble collapse on material ablation, i.e. cavitation erosion, however is under discussion. Takada et al. [80] showed that the bubble collapse also seems to play a role during LAL. They found cavitation erosion pits of similar morphology as in this work and interestingly, these pits were not produced once the bubble collapse was cushioned by increasing the vapor pressure. In our work material is only removed by the cavitation bubble collapse, thus the contribution of the bubble collapse to nanoparticle production can be estimated by comparing the volumetric erosion rate of cavitation to the laser ablation rates in LAL based nanoparticle production. Letzel et al. [48] and Streubel et al. [78] report ablation volumes per laser pulse, averaged over the first hundred pulses, of $3.05\cdot 10^{-13}\;m^{3}$ (nanosecond laser with silver substrate), and $2.58\cdot 10^{-17}\;m^{3}$ (picosecond laser with copper substrate), respectively, and maximum bubble radii of $R_{\max}=1655\;\mu$m and $R_{\max}=80\;\mu$m respectively. Normalization of the eroded volume on $R_{\max}^{3}$ implies that the cavitation collapse contributes to an amount of 14 % to 18 % of the ablation volume seen in LAL applications. This can be considered as a lower boundary as in the present work we aimed to achieve a highly repeatable bubble collapse resulting in the collapse to converge to the same position at the substrate. In LAL this repeatability of the collapse asymmetry usually is not crucial, instead it is optimized for maximum erosion.

In laser lithotripsy an optical fibre is used to deliver an infrared laser pulse to fragment and erode kidney stones [39]. Besides direct photothermal ablation, a cavitation bubble is formed between fibre output and stone. Recently, it was shown that for low repetition rates (⪅20 Hz) and pulses of duration shorter than 100μs, instead of photoablation cavitation damage is the dominant erosion mechanism [30], [14]. Interestingly, in these works the damage patterns on brittle kidney stone phantoms resemble the ones presented in this work and also show a critical stand-off sensitivity. This suggests a similar energy focusing mechanism at work, however, in those situations the bubble dynamics are more complex due to the additional rigid boundary from the fibre tip.

Recently, advanced strategies of fluid structure interaction have been developed that couple single bubble dynamics and their violent impact on boundaries with the aim to predict cavitation erosion [60], [13], [70]. For the same purpose, multiscale approaches for large scale technical flows are emerging that in addition couple large flow and pressure fields to single bubble models and their interaction with boundaries [31], [56]. These models require an understanding and accurate description of the cavitation induced erosion mechanisms. The present work may already allow improving the present erosion models, for example by accounting for the distance of the bubble from the boundary. Yet, we also acknowledge that asymmetries such as the presence of nearby bubbles or a boundary layer flow in large scale flows will affect the individual torus collapse and thus its erosiveness. To include these effects, the present experiment could be extended, e.g. by creating multiple bubbles, superimposing a flow, etc. Interestingly, the size and shape of the erosion damage found in technical relevant systems, for example, see the pits and craters in Crum [15], Bailey et al. [2], Haosheng and Shihan [28], Fernandez Rivas et al. [19], Tzanakis et al. [85], is very similar to the present reported drilled like holes and is thus not material dependent, but a general feature. Thus, an erosion modelling based on the present results could help to improve the forecasting of material failure in cavitating flows. Benjamin and Ellis [5] explained that only very few collapses result in cavitation damage: “This fact [that only 1 of 30,000 collapses are erosive] gives good reason to frame an explanation for cavitation damage as depending on a rather crucial combination of conditions for individual cavities.” We may want to complete their sentence as follows: “It may be that conditions are just right (or just wrong, one should perhaps say) when…” …conditions for the self focusing are met. This is the case when the ring collapse propagates about sonically.

A reliable technique to detect erosive cavitation from acoustic measurements is currently not available. Even though cavitation noise has been studied and the occurrence of cavitation can be well detected from broadband noise or from a significant energy content in the sub- and ultraharmonic frequency spectrum, the acoustic assessment of the erosiveness of cavitation has only been partly successful [44], [66], [76]. Here, it is shown that the signal from an erosive bubble collapse features a particularly short rise time of the peak pressure on the order of $t_{\mathrm{rise}}=40$ ns, which suggests the necessity to monitor the broadband noise above the respective Nyquist frequency of 2/40ns=50 MHz. These frequencies are strongly damped in liquids and therefore should be measured very close to the site with suitable hydrophones.

The erosiveness of cavitation is drastically decreased when the regular jet threads the bubble. This is the case for intermediate stand-offs (0.4⩽γ⩽1.0). There, the regular jet is responsible for a large collapse ring radius, implying less energy concentration due to splashing and winding up of the splash sheet, which additionally fragments the bubble. As a result many local and largely independent micro-collapses of lower intensity are induced. Additionally, the regular jet drives a boundary layer flow separating the bubble from the solid surface, such that the collapses of the fragmented bubbles occur at some distance from the solid. The decisive effect of the regular jet on the collapse was previously mentioned by Benjamin and Ellis [5] and Philipp and Lauterborn [58]. The jet induced splitting was studied numerically in rotational symmetry [94] and analytically by Brennen [9].

The regular jet impact itself is not responsible for erosion, even not through fatigue effects in any of the metallic materials tested.

The damage in the intermediate stand-off can clearly be attributed to the second collapse which is in accordance with previous works [73], [82], [58], [33], [18], [27]. This has been highlighted by Philipp and Lauterborn [58] who found that the second bubble collapse, which proceeds in toroidal form is the reason for the circular indentations. Similarly, Isselin et al. [33] concluded that the damage is produced from collective effects of microbubbles driven by the main bubble collapse. That damage consists of mainly an indentation only but again is a result of the energy focusing mechanism which however is less efficient during the second collapse.

## 5 Conclusions

Cavitation bubbles are only erosive if their first collapse occurs very close to the surface, i.e. only for γ≲0.2. The erosion mechanism involves a three-dimensional energy focusing through the shockwaves emitted during the bubble torus collapse. The torus collapses not all at once but progressively with a specific velocity. When this velocity approaches the shockwave velocity, a maximum shockwave amplification is achieved through constructive superposition of the emitted shockwaves. Then erosion is produced by a shockwave intensified collapse of the remaining bubble fragment. In that case already the collapse from one single bubble results in visible damage even on the hardest material (V2A steel) tested in this work.

The self-focusing effect here is strongest around γ≈0.1 and creates confined drilled-like holes in the substrate. For softer materials, the erosion rate per bubble is larger and the erosive stand-off range is also slightly larger. Here a less efficient self focusing is still able to produce some damage.

The regular jet with its velocity of about $100\;{\mathrm{ms}}^{-1}$, even though directed towards the solid, does not cause erosion of the studied samples. In contrary, it actually prevents erosion because it splits the torus. These tori are then larger and the jet induces instabilities that impede the self focusing. The erosive regime has a large overlap with the needle jet regime. The needle jet by itself is not causing erosion. Nevertheless, it is important as it prevents the formation of the regular jet results in a smaller and less fragmented bubble.

The results suggest a strategy to mitigate cavitation erosion: for a bubble collapsing at a wall, the regular jet must be stimulated to fragment the bubble. This could be done by appropriate surface structuring or introducing appropriate pressure gradients, for example via sonification. On the other hand, the findings imply strategies to intensify the collapse, as for example to achieve a more efficient nanoparticle production, laser-assisted material machining or for sonochemical reactions with more extreme conditions in the bubble interior. Here a small controlled asymmetry, purposefully introduced could be a promising strategy to achieve the most extreme energy concentration. Shock-resolving 3-dimensional simulations seem a natural way to understand which kind of asymmetries could lead to enhancing or prevention of cavitation erosion.

## Author contributions

FR designed and conducted the experiments and analysed the data, FR and CDO contributed to the interpretation of the data. CD contributed to surface analysis and performed the sample preparation. FR wrote the manuscript with input from all authors.

## Declaration of Competing Interest

The authors declare that they have no known competing financial interests or personal relationships that could have appeared to influence the work reported in this paper.

## Figures and Tables

**Fig. A.9:**
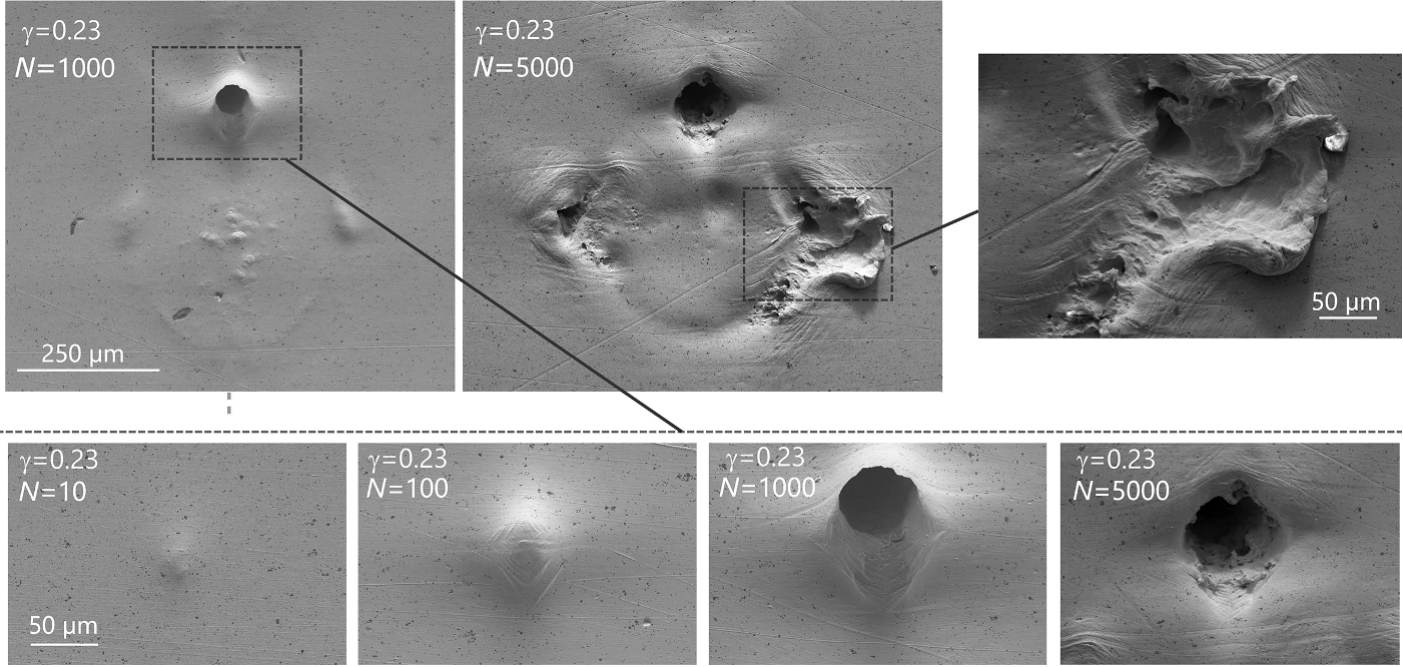
SEM images of the evolution of the damage pattern at fixed stand-off for increasing number of bubble impacts. Shown is the silver surface, and the stand-off was γ=0.23, i.e. in the transition regime of small erosion rates. *Top row:* Overview of the complete damage pattern at N=1000 and 5000, respectively, and magnification of the region of shear damage (the right image). Arrow: direction of incident laser beam and direction of buoyancy. ”BC” denotes the approximate position of the bubble center. *Bottom row:* Magnified view of the evolution of the drilled-like hole for N=10 to 5000 bubbles per position.

**Fig. B.10:**
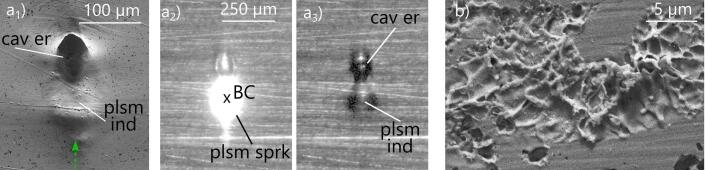
Surface damage on the silver sample. *a*_*1*_*):* Ex-situ SEM showing the cavitation induced erosion (“cav er”) and the indentation from the plasma seeding (“plsm ind”). *a*_*2,3*_*):* In-situ image of the surface with plasma spark (“plsm sprk”). The green arrow shows the directions of laser focusing and buoyancy, “BC” indicates center of the bubble that follows the plasma spark. *b):* Example of damage from direct plasma contact resulting in material evaporation (and partly re-deposition).

**Fig. C.11:**
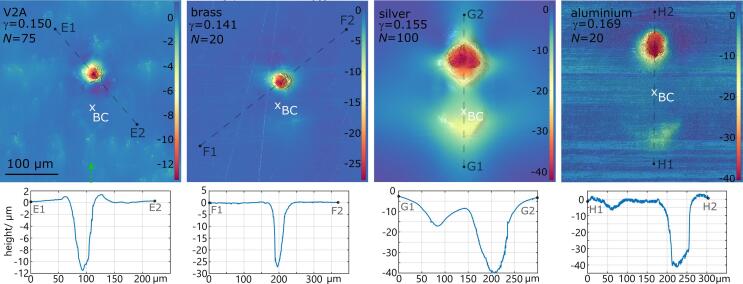
Comparison of damage patterns in the erosive stand-off regime on four metallic samples: V2A steel, brass, silver, and aluminium alloy. Each surface was exposed to N identical cavitation events at the same spot. All materials show erosion in the drilled hole like shape that occur off the symmetry axis of the bubble. The surface height is presented color-coded in micrometers. The green arrow in the first tile indicates the direction of laser beam focusing and buoyancy. ”BC” denote the approximate bubble centers. Illustrative profiles between the points $\overset{‾}{E_{1}E_{2}}$, $\overset{‾}{F_{1}F_{2}}$ etc. are presented in the lower column.

**Fig. D.12:**
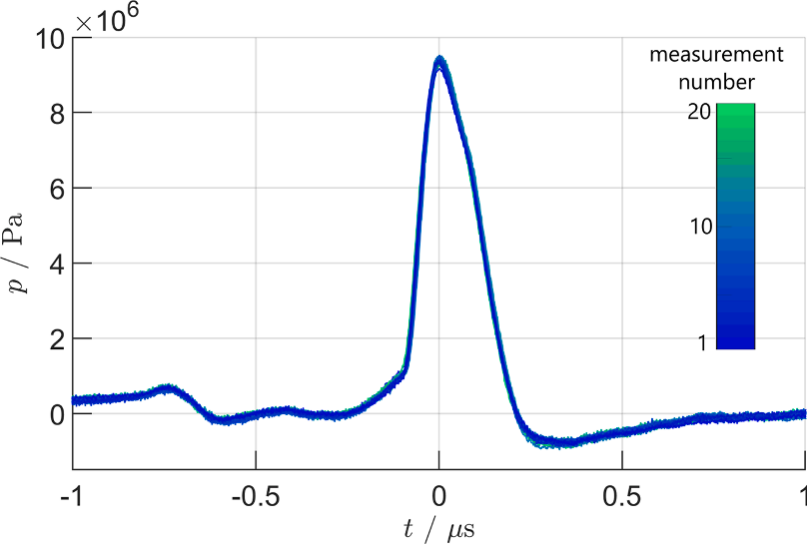
Dependence of the collapse pressure with increasing surface damage. Shown are the acoustic signatures of all subsequent N=20 bubbles during the evolution of the surface damage shown in Fig. C.11 (aluminium). The dark blue curve corresponds to the bubble at pristine surface, the brightest green color to the bubble collapsing at the surface with the final damage. All curves lie on top of each other, which indicates that the overall collapse dynamics is not altered by the surface damage.

**Fig. E.13:**
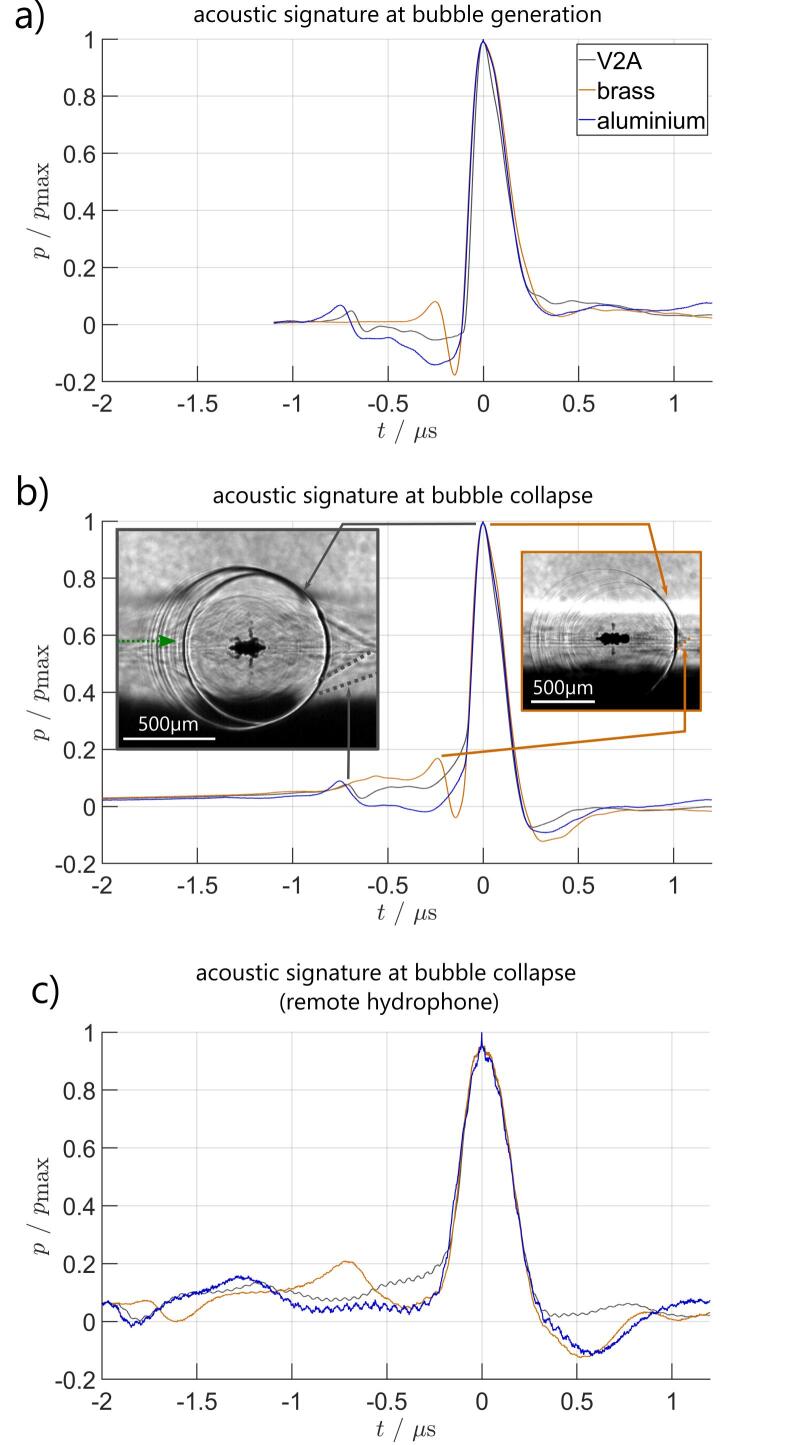
Comparison of acoustic signatures obtained with the PVDF hydrophone for three different materials at generation, and collapse. The hydrophone to plasma distance was $d_{\mathrm{hyd}}=4.9\;\mathrm{mm}$ for a and b. The tile c shows an additional probing from a more remote position of $d_{\mathrm{hyd}}\approx 8\;\mathrm{mm}$. All curves are normalized and aligned to their respective maximum and obtained by averaging several measurements within a stand-off interval of 0.06-0.12, i.e. in the erosive regime.

**Fig. F.14:**
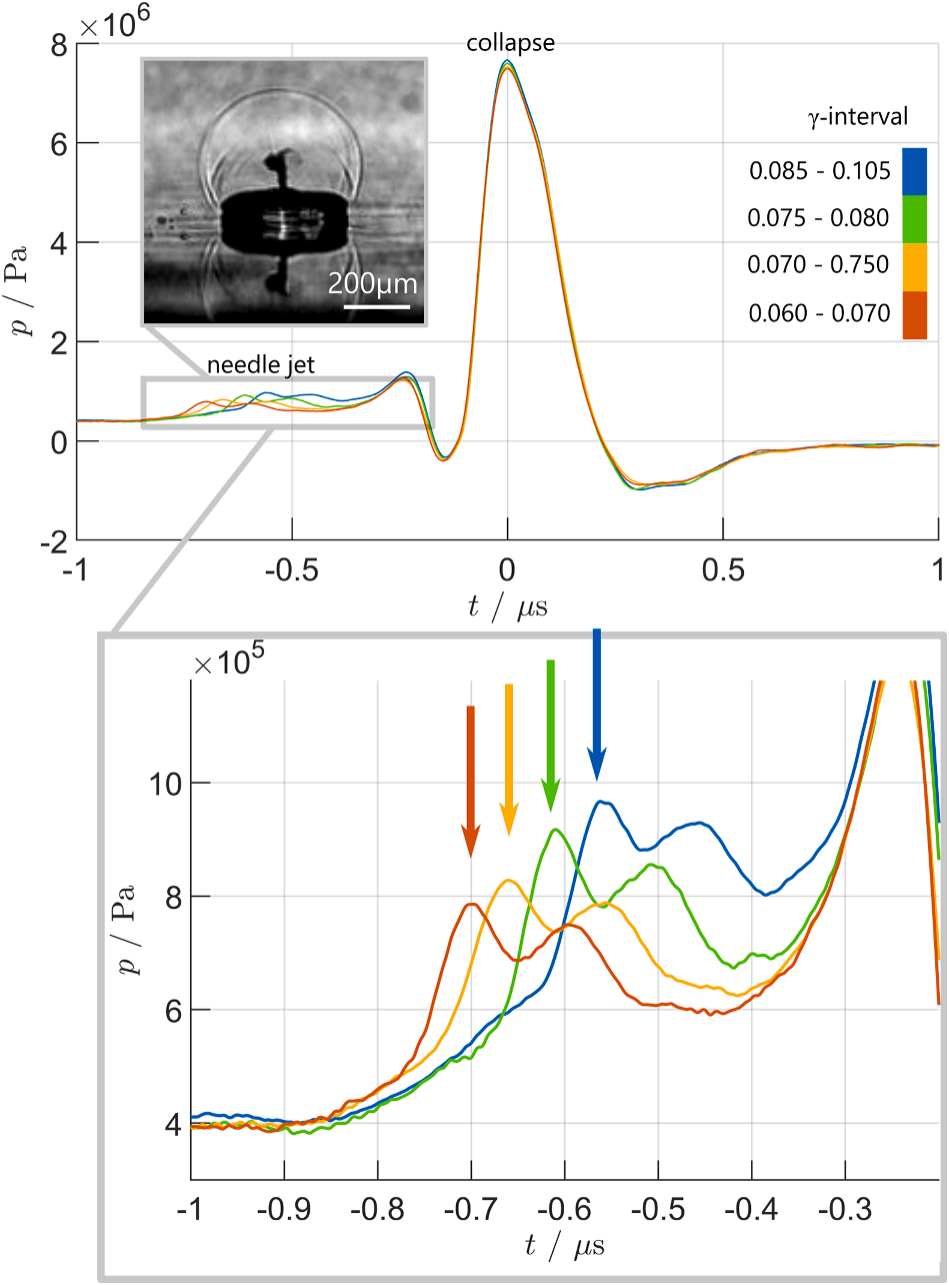
Acoustic signatures from needle jet formation. The instance of collapse is at t=0, the needle jet is formed a few hundred nanoseconds before, where the exact time is a function of the stand-off γ. Signatures were measured on the brass sample.

**Fig. G.15:**
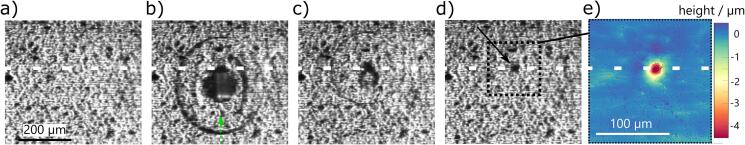
View of the V2A steel surface with bubble dynamics and in-situ damage. The mottled surface structure is typical for a rolled material. *a)* and *d)* show the pristine and eroded surface (see arrow), respectively. *b)* and *c)* show the location of the bubble shortly after seeding and the intensified collapse, respectively. In both cases a circular shock front is visible. The green arrow shows the directions of laser focusing and buoyancy. *e)* Color coded surface profile after 20 bubble impacts (maximum depth about 5μm).

**Fig. G.16:**
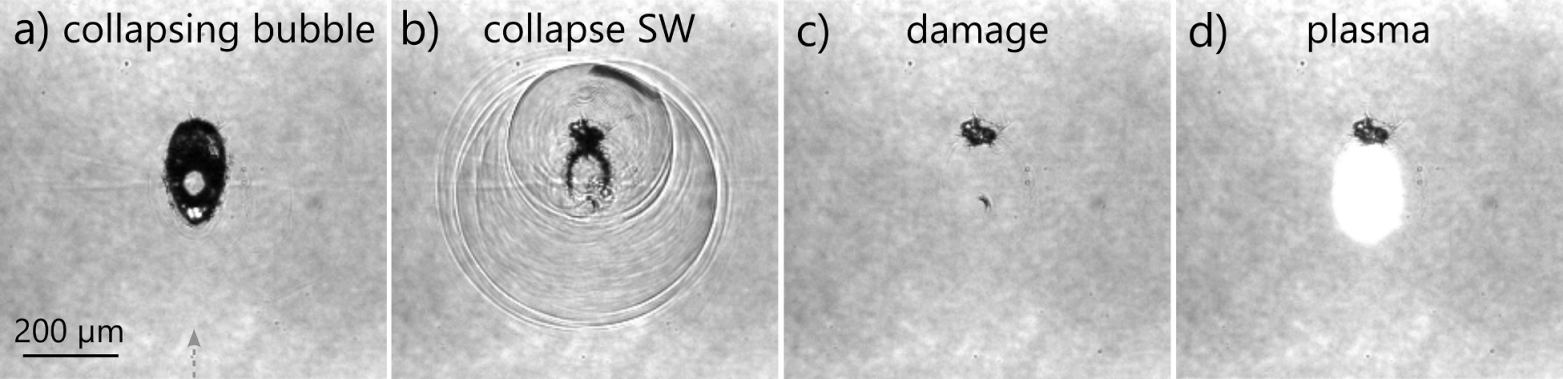
In-place observation of damage on the glass sample in the erosive regime in bottom view. *a)* shows the toroidal bubble before the collapse, *b)* captures the collapse shockwaves, and the green arrow shows the directions of laser focusing and buoyancy. The tile *c)* shows the damage under absence of any gas phase taken some seconds after b). The damage coincides with the emission centre of the shockwave from the intensified collapse in b). For reference, *d)* presents the plasma spark.

**Fig. H.17:**
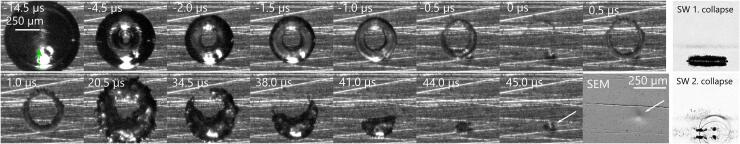
Non-erosive collapse at γ=0.38 with damage from the second collapse in top view. Times indicated with respect to the collapse frame. At t=45μs the damage already becomes visible in-situ and is further studied in the SEM image which shows the surface ex-situ after N=700 bubble collapses. The green arrow in the first tile indicates the directions of laser focusing and buoyancy. The damage is produced by a similar energy focusing mechanism as in the erosive stand-off regime but this time with less efficiency and during the second collapse. It now results only in the indentation without erosion, studied in Fig. 2.

**Fig. I.18:**
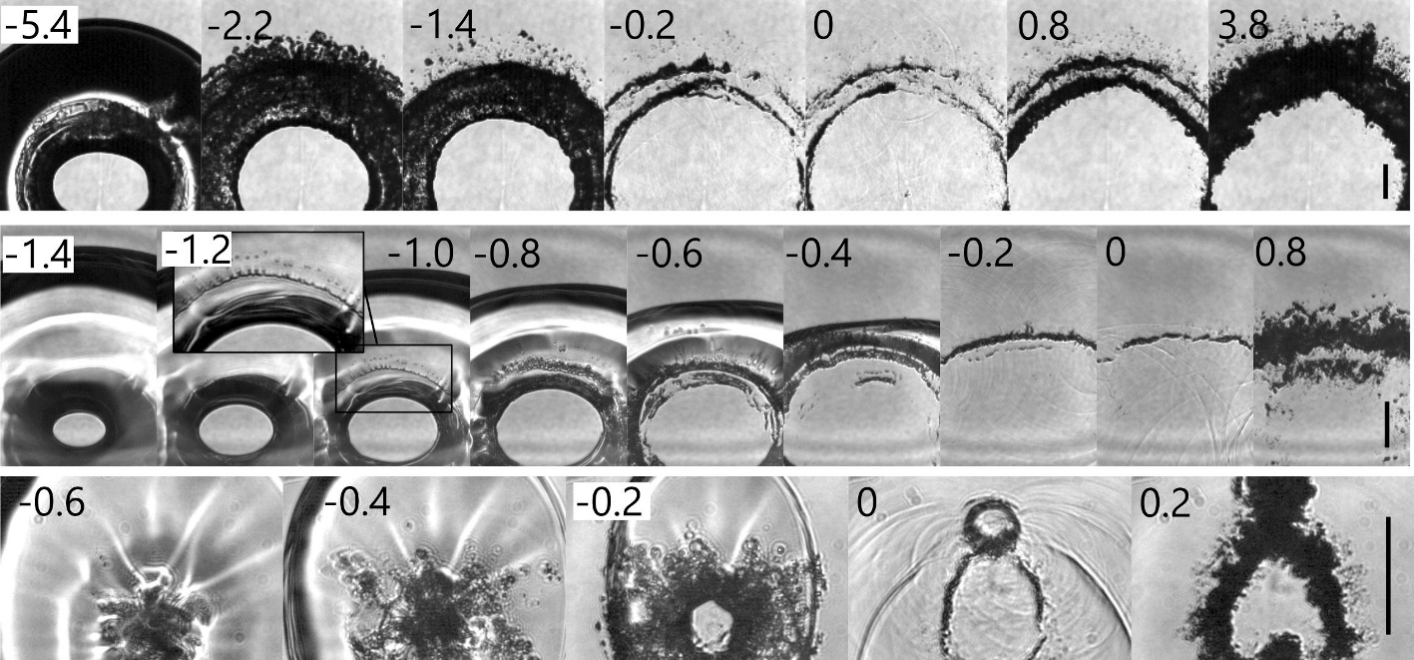
Jet impact, splashing and bubble fragmentation for three different stand-offs: from top to bottom, non-erosive, weakly erosive, and erosive case with γ=0.57, γ=0.24, γ=0.12, respectively. The time is indicated in each frame in microseconds, where the frame of minimum bubble volume is set to t=0. The scale bar in the last of each frame series denotes 100μm implying different magnifications for the three stand-offs. The inset in the middle series at t=-1.0μs is a zoomed in view of the instability of the splash sheet inside the bubble which results in internal droplet generation with a wavelength of the ripple of the order of 7μm.

## Appendix A Dependence on number of bubbles

Fig. A.9 demonstrates for γ=0.23, i.e. the stand-off where the bubble becomes weakly erosive, the evolution of the damage as a function of the number of bubbles *N*. The top row shows overviews for N=1000 and N=5000. In the latter case damage along the ring has manifested more clearly with some of the material being sheared radially outwards from the bubble center. In the shearing region, the material is heavily deformed and partially eroded. Peripherally, some waviness was introduced into the surface. The evolution of the eroded hole in the bottom row shows already at N=10 a characteristic mark that develops into the hole. At N=100, erosion becomes evident; the pit exhibits break-off edges along a rather rhombic outline which we connect with the crystal structure. The absence of polishing particles in the pit is a clear indicator for erosion. At N=1000 the hole has developed, and after 5000 bubble collapses the inner walls of the hole seem frayed out and material has piled up above the hole in the upper part of the picture.

## Appendix B Material damage from plasma seeding

The bubble seeding with the laser may also result in some damage on the substrate, which needs to be carefully separated from the cavitation induced damage, examples are shown in Fig. B.10. In a_1-3_) besides the cavitation induced erosion (“ce”), there is an indentation from the blast wave when the cavitation bubble is created. In-situ it becomes visible as darker region from the surface that is inclined towards the depression such that the reflected light does not hit the camera sensor anymore. Interestingly this indentation does not lead to material loss and *per se* is not erosion. Its location correlates with the plasma spark (“plsm sprk”) and only appears for the smallest stand-off distances within the erosive regime. Among the different materials studied, the indentation is deepest for the silver crystal. To reduce this effect and be able to also study smaller stand-offs within the erosive regime we used for the erosion experiments an improved focusing that decreases the size of the plasma. If the locations of the plasma induced erosion and cavitation induced erosion were too close to each other to distinguish them then the data was not evaluated. Fig. B.10b) Shows an extreme example of damage from direct plasma contact and focusing onto the substrate which resulted in evaporation, melting and re-deposition of the material. This effect was avoided in all evaluated measurements.

## Appendix C Comparison of material damage morphology on different metallic materials

While our focus lies on the understanding the origin of cavitation erosion, we also like to show that the material is not affecting the general shape of the pattern but only the rate at which erosion progresses. Fig. C.11 shows the surface damage in the erosive regime for a stand-off γ≈0.15 (standard deviation at each sample <0.014) for four materials of different Vickers hardness, namely aluminium (65HV5), brass (72HV5), and stainless steel V2A (167HV5). All materials show primary erosion in the shape of a single hole that does not coincide with the projected bubble center. Smaller differences concern the central material compression from bubble seeding observed only in the case of silver as well as the faintly visible ring structure in the case of the softest material (Al).

## Appendix D Repeatability of bubble collapse during progressive surface damage

Fig. D.12 shows the evolution of the pressure signature from 20 bubbles subsequently generated at the same sample position to study the evolution of the pressure signature with increasing damage. We chose the “worst-case” scenario, i.e. the case of largest eroded volume in Fig. 3 which is found for aluminium, the softest material. There, already after the 20 bubble collapses a significant damage is produced shown in Fig. C.11 (rightmost tile). As the damage is progressing, the pressure signatures virtually stay identical, indicating that the surface damage does not influence the overall bubble dynamics. This is also confirmed by the high-speed imaging. The reason becomes apparent when comparing the opening radius of the eroded hole to $R_{\max}$ which is about 2%. In addition, the curves confirm the repeatability of the bubble generation. Only when the damage extends on a larger surface, as with the sheared regions in Fig. A.9, the bubble dynamics eventually may become affected.

## Appendix E Acoustic signatures at different materials

In Fig. E.13 we compare the acoustic traces for different materials. The data is taken with the PVDF hydrophone as it is more sensitive in particular to the waves emitted from the solid because it has a less directional sensitivity than the optic hydrophone. To understand the origin of the peaks, it is helpful to also look at the curves from bubble generation (Fig. E.13a), in which case we omit the first data points as they contain electric noise from the laser control by its high voltage Q-switch operation but no acoustic signal. All curves are horizontally aligned and vertically normalized to their respective maxima which are of similar amplitude for the three materials for generation as well as for the collapse (order of 7.5 MPa normalized to 700μm probing distance). The central peaks at generation and collapse are of essentially similar shape and width in all three images. The main differences concern the precursor waves starting from about t=-1μs, and from around t=-2μs, respectively, at the more remote probing position. They stem from the part of the shockwave that travels partly through the solid until it reaches the hydrophone. The precursor occurs at a similar delay in the case of aluminium and V2A-steel as their respective wave propagation velocities are similar and significantly larger than the one of water. The approximate values for longitudinal and shear propagation velocity, denoted as $c_{l}$ and $c_{s}$, respectively, are: $c_{l}^{V2A}=5790\;{\mathrm{ms}}^{-1},c_{s}^{V2A}=3100\;{\mathrm{ms}}^{-1},c_{l}^{\mathrm{Al}}=6420\;{\mathrm{ms}}^{-1},c_{s}^{\mathrm{Al}}=3040\;{\mathrm{ms}}^{-1}$, while for brass: $c_{l}^{\mathrm{brass}}=4700\;{\mathrm{ms}}^{-1},c_{s}^{\mathrm{brass}}=2000\;{\mathrm{ms}}^{-1}$. The insets in b) show the collapsed bubble as black object in the center, taken some hundreds of nanoseconds after the collapse at the steel on the left and the brass sample on the right. The shock fronts travelling in water appear as circles where the lower semicircles are seen as reflections from the substrate (the boundary is located horizontally). The circular shock front is responsible for the central pressure peak. In addition both images show linear fronts from Schmidt head waves, these are generated by shockwaves travelling in the solid as transversal and leaky Rayleigh waves that couple part of their energy into the liquid, as they propagate along the solid surface. In the case of V2A, the Schmidt head wave front has a larger angle towards the solids surface normal and an increased distance to the circular shockwave. This is because $c_{s}^{V2A}$ is significantly larger than $c^{H2O}$, i.e. the waves in the solid propagate pseudo-supersonically with respect to water. As a consequence, the shockwave travelling through the solid reaches the hydrophone, which is located in horizontal direction left in each image, before the shockwave that travelled entirely through water. The head wave peak is further advanced in the case of aluminium because $c^{\mathrm{alu}}\;>\;c^{V2A}\;>\;c^{\mathrm{brass}}$. The lower speed of sound in brass however, lets us investigate the shockwave emission during needle jet formation in Fig. F.14b as the Schmidt head wave is sufficiently slowly propagating to not interfere with the former.

Further, it can be noted from comparing the right part of the central peaks (positive times) at seeding and collapse that only in the later case a rarefraction phase (negative pressure) is present, likely due to reflections of the shockwave at the bubble gas phase which induces a phase shift. An indication may be the finer structure of further shockwaves modulated within the main circular shockwave in the insets resulting from reflections and collapses of bubble fragments.

In Fig. E.13c), the pressure transient measured at a more remote location is shown. There, the pressure amplitudes have decreased, seen here by the noise ripple, and the peaks have dispersed, i.e. show larger widths. In addition further precursors from reflections occur and the separation between central peak and precursor has increased due to the larger propagation distance.

## Appendix F Shockwaves from needle jet generation

In Fig. F.14 the acoustic signatures of needle jet formation and bubble collapse, are shown for different stand-offs. The central peaks at t=0 belong to the collapse shock and the precursor peaks in the inset belong to the needle jet formation seen at frames -600 ns and -400 ns in Fig. 4a). The peaks around t≈-200μs followed by a tension phase stem from a Schmidt head wave. While the central peak remains virtually unchanged, for increasing stand-off the peak from needle-jet formation shifts temporally towards the instance of collapse. This behaviour has been predicted numerically by Lechner et al. [47]. In addition, the peak amplitude decreases and seems to fade out between γ≈0.12 and 0.20, after which it is not observed anymore (not shown). To reduce noise, several measurements are aggregated by averaging within each stand-off interval and the data is smoothed by convolution with a Gaussian window (FWHM 7ns).

## Appendix G Simultaneous observation of bubble dynamics and damage

While the damage on the substrate can already be seen in side view due to some microscopic elevations, we confirm the position of the surface damage and in particular of the eroded hole with respect to the bubble from simultaneous observations of both. Therefore, the high speed camera is pointed in top view onto the substrate and a beam splitter is employed to allow for inline illumination (see Fig. 1c). Fig. G.15a shows a view of the pristine V2A steel surface, and Fig. G.15b and c show the bubble during generation, and collapse, respectively. Comparison of c, d, and e reveals that the erosion appears at the location from where the shockwave of the intensified collapse is emitted from. While our focus is not the study of damage on the glass sample, we would also like to show that damage on glass is again produced at the spot of intensified collapse, and not in the jet impact region, see Fig. G.16.

## Appendix H Damage from second bubble collapse

In Fig. H.17 we are looking from top onto the collapsing bubble and use the silver sample as a mirror in an effort to correlate the location damage with the bubble dynamics. The particular bubble dynamics in this figure is recorded after N=700 similar bubbles have collapsed in similar manner. As a result, a small indentation at the lower right part of the frames is visible and is indicated with a white arrow at t=45μs and in the SEM-image. This indentation is outside the ring where the first bubble collapses, yet is exactly at the spot where the now disturbed torus collapses a second time. The rightmost tiles show the shockwave fronts at first and second collapse, respectively, from a similar bubble. While at the first collapse many weaker shockwaves are emitted, now the second collapse comes with a stronger, localized shockwave emission. Thus, the second collapse here involves again an energy focusing mechanism similar to the one observed during the first collapse in the erosive regime. It is interesting to note that the collapse does not take part in direct contact to the solid as the side view reveals and results only in an indentation as the SEM image shows.

## Appendix I Role of jetting on cavitation erosion

Here we look into some of the fluid mechanics induced by the jetting as a function of the stand-off distance with respect to the erosiveness of the collapse. Then we discuss the direct role of the jet with respect to erosion.

The imaging series in Fig. I.18 compares the jet impact, splashing, and the later torus collapse for decreasing stand-off distance from γ=0.57 to γ=0.12. At a larger stand-off distance, top row in Fig. I.18, the regular jet impacts earlier with respect to the bubble collapse, ΔT=-5.4μs. This results in splashing of a rather thick sheet that splits the torus into two tori. Due to the early jet impact, the resulting tori are of rather large diameter. In the transition stand-off region, here γ=0.24 where mild erosion is found (middle row in Fig. I.18) the splash sheet is thinner and the generated droplets are smaller. The inset magnifies the sheet and reveals the instability on the rim of the splash. Fast droplet impacts on the free surface of the collapsing torus are the result of this pinch-off dynamics. Here the outer bubble torus is already in the late collapse state when the jet impacts, ΔT=1.4μs, thus the diameter of the torus is noticeably smaller. Even though at this stand-off the needle jet does not form, we still find the drilled-like holes erosion pattern observed in the smaller stand-off needle jet regime, however the erosion rate is much smaller.

In the erosive regime, bottom row in Fig. I.18, the bubble is penetrated only by the needle jet. Its smaller diameter and lesser fluid transport together with the fast collapse of the outer bubble wall mutually result to lesser fragmentation of the bubble prior collapse. Here, the needle-jet impact and the bubble collapse almost coincide within an interval of less than 400 ns.

The frames of the rebound stage, t>0, for the three stand-off distances shown in Fig. I.18 reveal a re-expansion of fragmented rings consisting of microscopic, individual bubbles. Please note their different timing and magnification.

The dependence of the torus collapse radius $R_{\mathrm{rc}}$ as a function of stand-off distance is presented in Fig. I.19. It is measured in the image of minimum gas volume in high-speed recordings taken in side view. The recordings were taken at 1 million fps, thus introduce some uncertainty in determining the moment of collapse. To estimate the uncertainty that comes from the lower frame rates in determining the torus width, one can use the averaged bubble wall velocity in parallel to the boundary of about $50\;{\mathrm{ms}}^{-1}$ measured around the collapse frame. The uncertainty in the determination the instance of collapse is 0.5μs therefore the error in $R_{\mathrm{rc}}$ is ±50μm or $R_{\mathrm{rc}}/R_{\max}\approx 7\%$.

The largest torus diameter is found for γ≈0.6 with a normalized torus collapse radius of 0.57. This is in accordance with previous measurements [82], [50], [64]. In addition to those measurements, here we provide data for γ approaching zero. For the largest tori, the time interval between jet impact and collapse is highest. This time interval decreases with smaller stand-off distance, i.e. from γ=0.6 towards γ=0.2. This behaviour has been predicted and analysed in detail by [47] through numerical simulations. At γ≈0.2 the torus radius decreases abruptly before it levels off for γ<0.2. This abrupt change is a result of the qualitatively different bubble dynamics, i.e. the needle jet regime. Careful inspection of the data may suggest that there exists a minimum around γ=0.17, which would correlate well with the most erosive stand-off. As for smaller torus radii the initial bubble energy gets concentrated on a smaller volume, we expect for smallest tori highest energy concentrations.

While we have shown that the torus collapse results into damage, the regular jet impact itself does not cause damage. This can be supported by estimation of the stresses during jet impact and relating them to the material parameters. The stagnation pressure of the regular jet with velocity $v_{\mathrm{rj}}\approx 100\;{\mathrm{ms}}^{-1}$ can last for longer times in the order of a few microseconds. However, its value is as low as $p_{\mathrm{rj}}=\frac{1}{2}\rho v_{\mathrm{rj}}^{2}=5$ MPa, where $\rho =1000\;\mathrm{kg}\;m^{-3}$ is the mass density of water. This is far below the tensile strength of most metals or metal alloys and even far below their yield strengths. For silver, tensile and yield strength are 110-340 MPa and 55 MPa, respectively [74]. Besides the stagnation pressure, a water hammer pressure is generated, i.e. the pressure in acoustic approximation that is generated at the instance of jet impact onto the solid. It is linearly dependent on the jet velocity and can be estimated by $p_{\mathrm{rj}}=\frac{1}{2}\rho {\mathrm{cv}}_{\mathrm{rj}}=74$ MPa, which is still not enough to cause plastic deformation in most cases. Besides, the experimentally occurring water hammer pressures are likely much smaller as the jet is cushioned by a thin liquid layer that resides between bubble and solid [63]. In addition, water hammer pressures are only of short duration of the order of 10 ns [97]. Accordingly, in our acoustic measurements we did not detect significant water hammer pressures from regular jet impact which is in agreement with numerical predictions [92].
